# Comparative Three-Dimensional Finite Element Analysis of Stress Distribution and Deformation in Endodontically Treated Maxillary Premolars Restored With Composite and Different Fibre Reinforcement Strategies

**DOI:** 10.7759/cureus.110079

**Published:** 2026-06-01

**Authors:** Gauri Arora, Sarita Gill, Pradipkumar Damor, Shubham Goyal, Dipendra Karndhar

**Affiliations:** 1 Division of Conservative Dentistry and Endodontics, Army Hospital Research and Referral, Army Dental Centre Research and Referral, New Delhi, IND; 2 Division of Conservative Dentistry and Endodontics, Centre for Dental Education and Research, All India Institute of Medical Sciences, New Delhi, New Delhi, IND

**Keywords:** composite restoration, cuspal deflection, fibre reinforcement, finite element analysis, horizontal fibre post, ribbond, stress distribution

## Abstract

Background: Large mesio-occluso-distal (MOD) cavities significantly weaken posterior teeth, increasing the risk of deformation and fracture. Fibre reinforcement techniques have been proposed to improve the biomechanical behaviour of composite restorations.

Aim: To evaluate and compare the stress distribution and deformation patterns of MOD-restored premolars using direct composite alone, horizontal fibre post, fibre-reinforced direct composite, and a combined reinforcement technique.

Materials and methods: Four three-dimensional (3D) finite element models of a maxillary premolar were generated and restored with different reinforcement strategies. Static occlusal load of 300 N was applied, and Von Mises stresses, deformation values, and stress localisation were analysed. Stress patterns were compared across enamel, dentin, periodontal ligament, restorative materials, and reinforcement fibres.

Results: Direct composite exhibited unfavourable stress concentration at the buccal and palatal cusp inclines and cervical region. Horizontal fibre posts effectively splinted cusps and redirected stresses away from marginal ridges. Ribbond fibre resulted in more uniform stress distribution with reduced cusp stress. The combined technique produced the lowest deformation (3.721 µm) and most favourable stress distribution, with minimal stress at cusp tips, cervical regions, and internal line angles.

Conclusion: Fibre reinforcement improved stress distribution in MOD restorations, with the combined technique providing the most favourable biomechanical outcome. Incorporating Ribbond and horizontal posts may enhance restoration longevity and reduce fracture risk in structurally compromised premolars.

Clinical significance: Fibre-reinforced techniques offer a viable option for restoring extensive MOD cavities, improving cuspal stability, and reducing stress-induced failure.

## Introduction

Endodontic treatment aims principally to eradicate pulpal infection, alleviate symptoms, and preserve the natural dentition; however, it inevitably alters the biomechanical behaviour of the tooth through changes in proprioceptive feedback, dentin moisture content, and structural continuity [1,2]. Long-term outcomes of endodontically treated teeth (ETT) are therefore strongly influenced by the quality of the post-treatment restoration. Endodontic causes accounted for only 7% of ETT failures, while 36% were caused by restorative complications. This highlights the importance of coronal integrity in determining survival [3]. Premolars are especially vulnerable to structural weakness due to their steep cervical dimensions, sharp cuspal angles, and comparatively thin buccal and lingual walls [4]. Loss of marginal ridges, as occurs with MOD preparations, eliminates essential dentin bulk and disrupts cuspal continuity, resulting in a profound reduction in fracture resistance and increased tensile stress within the cervical and occlusal regions [5]. Although direct resin composite remains a widely employed post-endodontic restorative modality with favourable clinical success rates, limitations such as polymerisation shrinkage stress, suboptimal flexural reinforcement, and inadequate support of severely weakened structures can compromise biomechanical performance [6].

Fibre-reinforced composite systems, particularly ultra-high-molecular-weight polyethylene fibres (e.g., Ribbond®), have been introduced to enhance internal stress modulation. Their high toughness, anisotropic behaviour, and dentin-compatible elastic modulus enable them to function as internal splints, dissipating occlusal forces and limiting crack propagation. Fibre-reinforced composite (FRC) plays a crucial role in enhancing the fracture strength of restorations for both endodontically treated and non-ETT, as well as in improving microleakage and marginal integrity of the restorations [7]. Similarly, horizontal or transfixed fibre posts have been proposed as a minimally invasive method to mechanically couple buccal and lingual cusps, thereby countering cusp divergence - one of the dominant failure modes in MOD-restored premolars [8,9]. However, existing evidence remains inconsistent, and data specific to premolar morphology are sparse.

Finite element analysis (FEA) has emerged as a critical tool for quantifying stress trajectories within restored ETT, enabling controlled evaluation of material behaviour, interface mechanics, and the effect of reinforcement design on stress distribution [10]. Given the heterogeneity in fibre types, orientations, and post configurations across published studies, a lack of consensus remains regarding the most effective reinforcement strategy for structurally compromised premolars. Hence, the present study was planned to evaluate and compare the biomechanical behaviour of endodontically treated maxillary premolars with standardised MOD cavities restored using four different restorative strategies: (1) direct composite restoration, (2) composite supported by a horizontal transfixing glass fibre post, (3) composite reinforced with Ribbond polyethylene fibre, and (4) a combined approach using both Ribbond and a horizontal fibre post-using three-dimensional FEA (3D-FEA).

## Materials and methods

FEM generation

A permanent, non-carious maxillary first premolar tooth extracted for orthodontic treatment was selected for the present study. The tooth was cleaned, and any visible calculus, if present, was removed. It was inspected to confirm the absence of fractures and resorption defects. A high-resolution cone beam computed tomography machine (Planmeca ProMax 3d MID; Planmeca, Helsinki, Finland) running in endodontic mode at 90 kV and 12 mA with a voxel dimension of 75 μm was used to scan the chosen premolar. Enamel, dentin, pulp chamber, periodontal ligament (PDL), cortical bone, and cancellous bone were segmented to obtain a complete anatomical model suitable for finite element analysis (FEA). To simulate an endodontically treated tooth, an endodontic access cavity was prepared in the reconstructed model, followed by virtual root canal instrumentation and obturation with gutta-percha. The obturated canal space and pulp chamber were incorporated into the FEM, and the corresponding material properties were assigned according to published literature. A 200-μm PDL layer was modelled around the root, followed by a bone block extending 3 mm apical to the cementoenamel junction (CEJ), consistent with previous FEA studies [11].

Preparation of standardised MOD cavities

Following reconstruction of the intact tooth, a standardised mesio-occluso-distal (MOD) preparation was created. The preparation retained 2.5 mm of buccal and lingual wall thickness at the height of contour, and the gingival floor was positioned 1.5 mm coronal to the CEJ, consistent with previous FEA studies. The same MOD cavity geometry was replicated across all experimental groups to ensure model uniformity.

Restorative configurations

**Figure 1:**
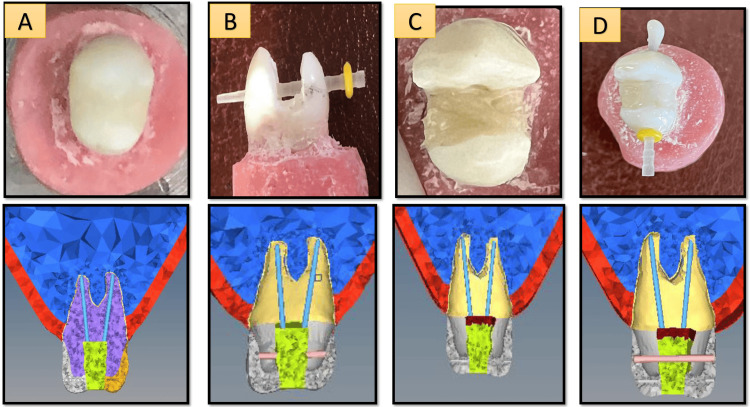
Clinical and corresponding finite element models of MOD-restored maxillary premolars with different reinforcement strategies (A) Direct composite, (B) horizontal fibre post, (C) Ribbond fibre, and (D) combined reinforcement, illustrating structural configuration and stress-modulating design. MOD: Mesio-occluso-distal

Four restored models were generated based on the prepared premolar (Figure 1).

Model 1 (Figure 1A): Direct Composite

The MOD cavity was restored entirely with nanohybrid composite resin incrementally without reinforcement.

Model 2 (Figure 1B): Horizontal Fibre Post + Composite

A transfixing glass fibre post was inserted buccopalatally through a pre-prepared horizontal channel to splint both cusps. The post was luted using flowable composite, followed by conventional incremental composite restoration.

Model 3 (Figure 1C): Composite + Ribbond Fibre

A 2-mm-wide polyethylene fibre (Ribbond) was embedded in the cervical third at the floor of the cavity using a 0.1-mm flowable composite liner, followed by incremental composite layering.

Model 4 (Figure 1D): Ribbond + Horizontal Fibre Post + Composite (Combined Group)

Both reinforcement strategies were combined: ribbond insertion at the base of the cavity, followed by a horizontal fibre post in the middle of the buccal and palatal walls, and composite restoration.

Meshing and setting material properties

**Figure 2:**
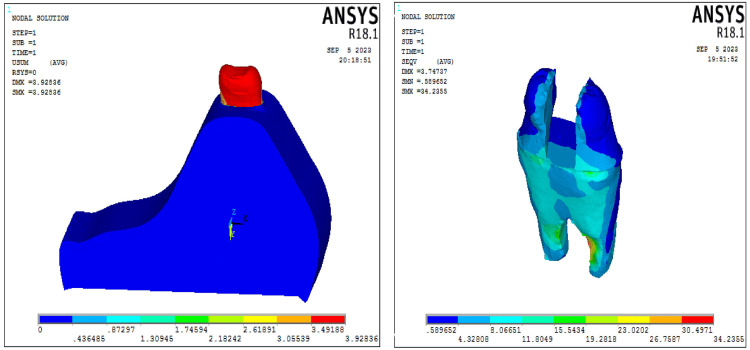
Construction of geometric models Mesio-occluso-distal (MOD) cavity on a permanent maxillary premolar computed using the elastic properties of individual structures after meshing.

All geometric models were imported into HyperMesh V11 for pre-processing and mesh generation (Figure 2).

All materials were assumed to be homogeneous, isotropic, and linearly elastic, consistent with validated FEA protocols. Elastic modulus and Poisson's ratio values were adopted from published literature (Table 1), including enamel, dentin, PDL, cortical and cancellous bone, composite resin, flowable composite, and fibre-reinforced components [12].

**Table 1:** Mechanical properties of materials Mechanical Properties of Materials

Material	Elastic Modulus (GPa)	Poisson's Ratio
Enamel	84	0.33
Dentin	18.6	0.30
PDL	0.0000689	0.45
Cortical bone	13.7	0.30
Cancellous bone	1.37	0.30
Composite resin	24.5	0.31
Flowable resin	13.5	0.39
Gutta-percha	0.14	0.45
Fibre (Ribbond)	33.1	0.22
Glass fibre post (transfixed)	37 (longitudinal axis)	0.27

Finite element analysis

Mechanical loading simulations were performed using ANSYS 2023 R1. A static vertical load of 300 N was applied to the buccal and palatal cusp inclines via a 5-mm spherical indenter, replicating functional occlusal forces. The load magnitude and distribution followed previously established protocols for premolar biomechanics [13]. FEA evaluated the stress distribution patterns, maximum von Mises (VM) stress, maximum principal stress (MPS), and cuspal displacement following load application across the four restorative configurations using ANSYS Workbench 2023 R1 software (ANSYS Inc., Canonsburg, PA). All values were tabulated and compared, and stress distribution patterns were analysed.

## Results

**Figure 3:**
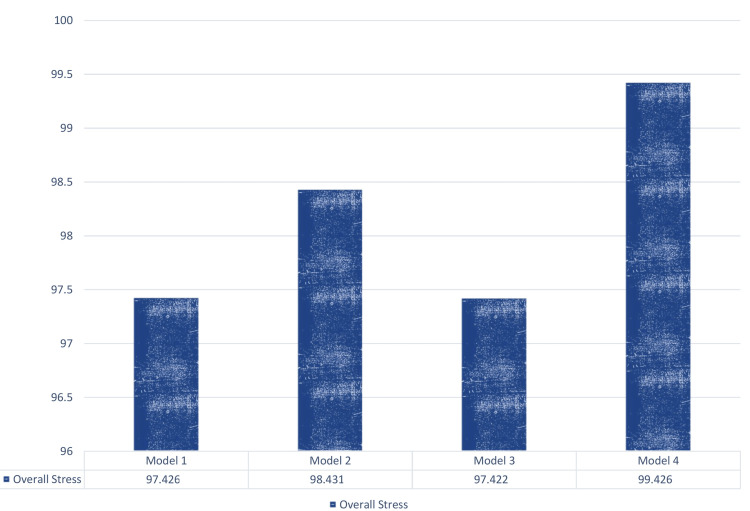
Overall stress comparison across models The overall von Mises stress values differed among the four models, with Model 4 exhibiting the highest stress bearing, while Model 1 and Model 3 showed the lowest overall stress bearing.

The mechanical analysis of the four finite element models revealed distinct differences in deformation behaviour and stress distribution patterns. The FEA findings are presented as colourimetric graphs. The overall VM stress values recorded for the models were as follows: 97.42 MPa for Model 1, 98.43 MPa for Model 2, 97.43 MPa for Model 3, and 99.42 MPa for Model 4 (Figure 3).

**Figure 4:**
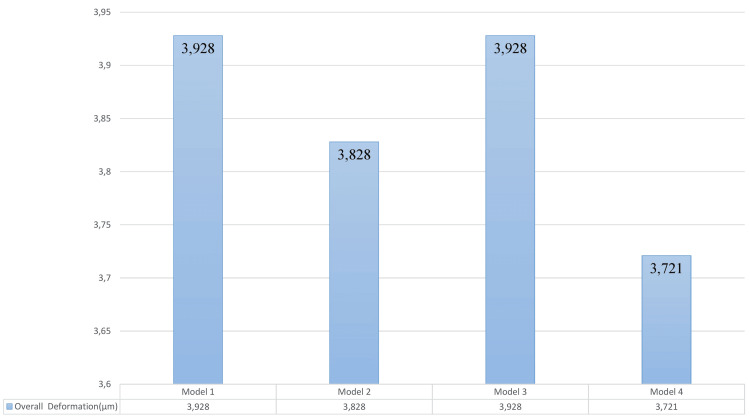
Overall deformation comparison across models The overall deformation varied among the four models, with Model 4 exhibiting the lowest linear deformation, while Models 1 and 3 showed the highest linear deformation values.

Lowest deformation was seen in Model 4 (3.721 µm) while showing the highest overall stress (99.43 MPa), suggesting a stiffer structural configuration with increased stress concentration, particularly within the enamel regions (59.37 MPa) (Figure 4).

Model 2 exhibited a slightly higher overall stress (98.43 MPa) and moderate deformation (3.828 µm), with the horizontal fibre post recording localised stress peaks of 9.06 MPa. Models 1 and 3 displayed comparable deformation values (3.928-3.993 µm) with similar overall stress levels (97.42-97.43 MPa). Model 3, which incorporated Ribbond reinforcement, exhibited localised stress values between 4.02 and 4.12 MPa, indicating internal stress absorption by the fibre layer.

**Figure 5:**
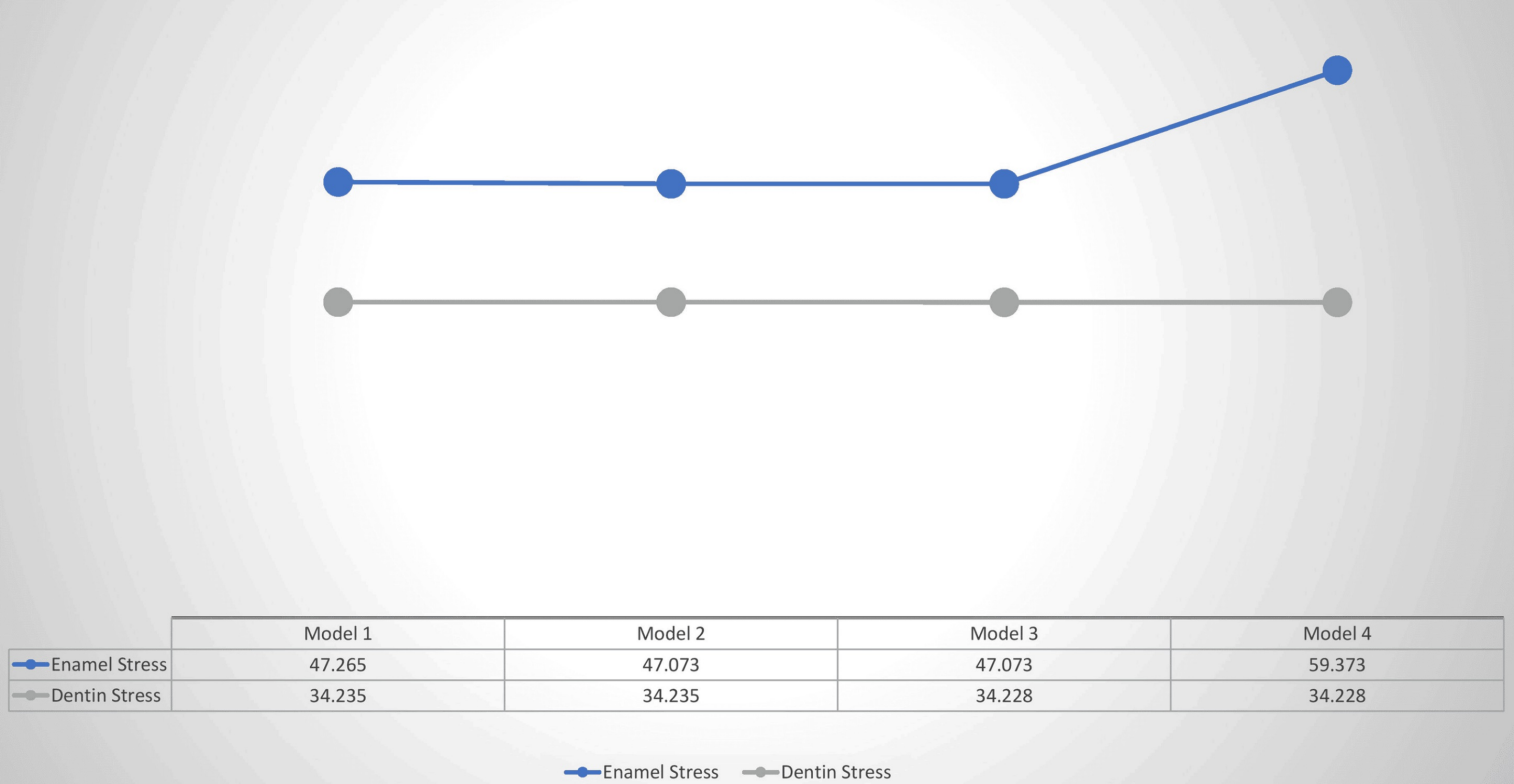
Overall enamel and dentin stress across models The von Mises stress distribution within enamel and dentin varied across the four models, with Model 4 exhibiting a marked increase in enamel stress compared to the other configurations, while dentin stress remained relatively consistent among all models.

Analysis of enamel and dentin stresses revealed distinct patterns among the four restorative models. Enamel stress values were comparable in Models 1, 2, and 3, with mean values of 47.26 MPa, 47.07 MPa, and 47.07 MPa, respectively. In contrast, Model 4 demonstrated a marked increase in enamel stress, recording the highest value of 59.37 MPa. Dentin stress values, however, remained relatively consistent across all models, ranging from 34.23 MPa to 34.29 MPa, with no clinically significant variation observed (Figure 5).

**Figure 6:**
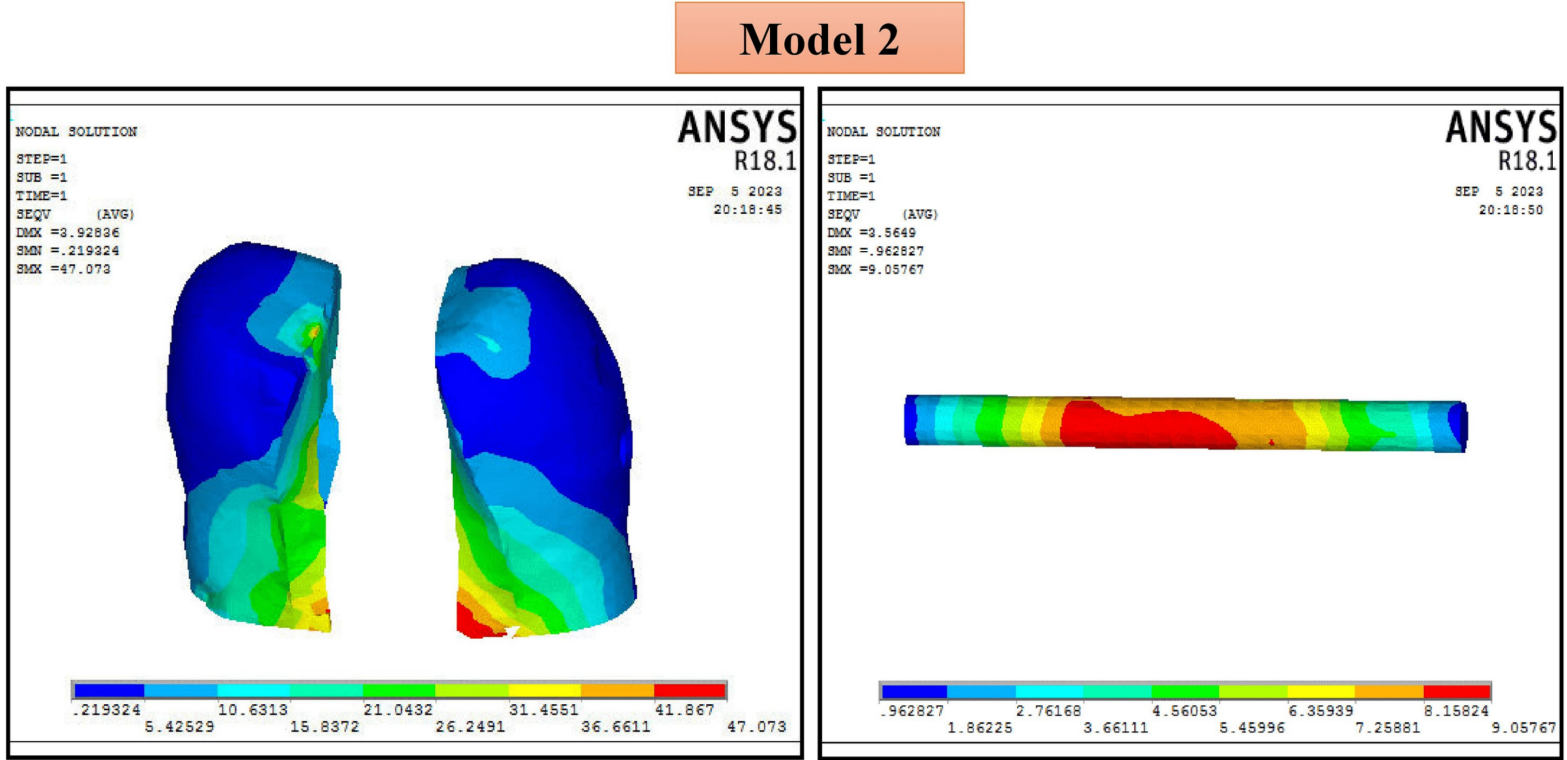
von Mises stress distribution and cuspal displacement due to vertical loading in Model 2 Stress concentration at the centre of horizontal fibre post when incorporated for restoring a mesio-occluso-distal (MOD) cavity. The colour bar is used to visualise the results of structural analysis by representing the intensity of mechanical stress, with warmer colours indicating high-stress areas and cooler colours indicating low-stress areas.

Stresses within cortical and cancellous bone, dentin, and periodontal ligament remained relatively consistent across all groups, whereas stresses in Gutta-percha and pulp were negligible or absent. Stress analysis performed for the individual restorative designs further clarified these patterns. Model 1 (direct composite) displayed distinct stress concentration at the buccal and palatal cusp inclines and the cervical region, reflecting unfavourable stress localisation and greater fracture susceptibility. Model 2 (horizontal fibre post) redistributed stresses more efficiently across the tooth structure, reducing peak stress values around marginal ridges (Figure 6).

**Figure 7:**
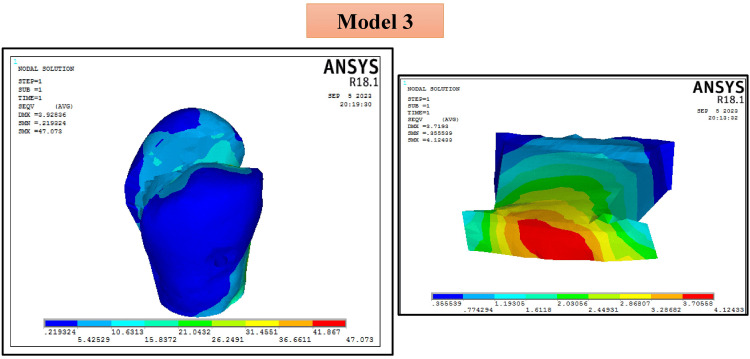
von Mises stress distribution and cuspal displacement due to vertical loading in Model 3 Stress on polyethylene fibre after cavity restoration.

Model 3 (fibre-reinforced composite) showed a more uniform stress distribution, with noticeably reduced stress intensity at both cusp tips (Figure 7).

**Figure 8:**
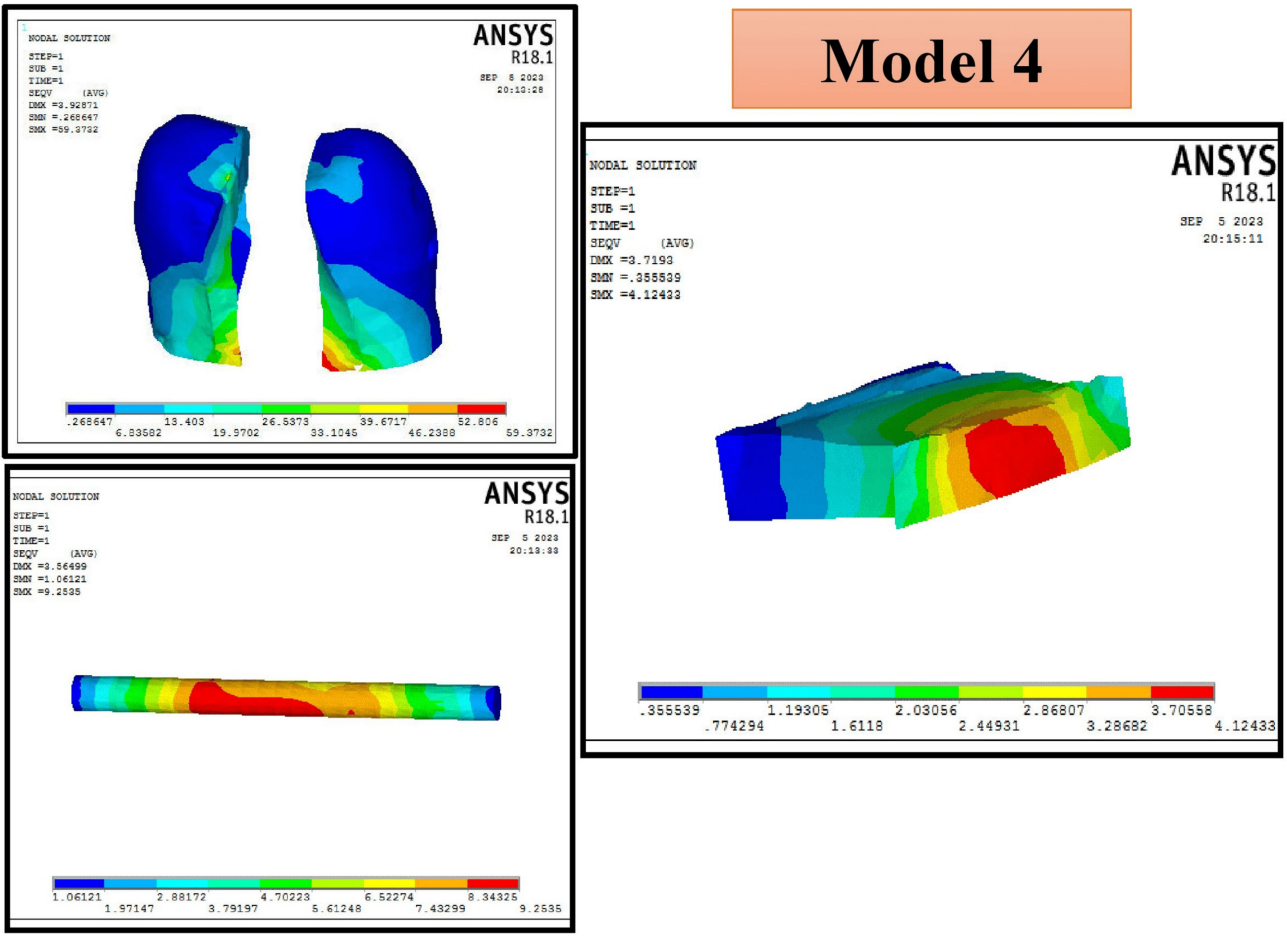
Von Mises stress distribution and cuspal displacement due to vertical loading in Model 4 Stress distribution on horizontal fibre and polyethylene fibre after combined restorative technique. The colour bar is used to visualise the results of structural analysis by representing the intensity of mechanical stress, with warmer colours indicating high-stress areas and cooler colours indicating low-stress areas.

Model 4 (combined technique) demonstrated the most favourable stress pattern, characterised by minimal stress concentrations at cusp tips, cervical margins, and internal line angles (Figure 8).

## Discussion

The restoration of ETT with extensive MOD cavities presents a significant biomechanical challenge due to the loss of marginal ridges and reduced dentin bulk, which predispose premolars to cuspal deflection and fracture [14]. The cuspal inclination of premolars increases their susceptibility to cusp fracture under occlusal force [15]. MOD cavities further reduce the tooth strength, rendering them vulnerable to fractures. Consequently, the long-term success of post-endodontic restorations is strongly influenced by the ability of the restorative approach to redistribute functional stresses and stabilize the remaining tooth structure. The present study aimed to evaluate and compare the biomechanical behaviour of endodontically treated maxillary premolars with standardised MOD cavities restored using different fibre-reinforcement strategies, by analysing stress distribution and deformation patterns through three-dimensional finite element analysis.

Previous investigations have validated the reliability of FEMs for assessing failure mechanisms in diverse materials [16]. The findings indicated a strong correlation between FEM simulations and corresponding experimental observations, supporting the use of FEM as a dependable analytical tool.

In the present study, a sound tooth model served as the reference standard for comparing stress distribution and assessing how closely the restored models replicated the biomechanical behaviour of an intact tooth. Comparing stress in tooth, enamel showed higher stress values than dentin across all models (47.07 > 34.2 MPa), reflecting its higher elastic modulus and brittle nature. In Models 1-3, enamel stress remained relatively similar (47.07 MPa), indicating limited stress modulation with direct composite or single reinforcement strategies. In Model 4, enamel stress was noticeably higher (59.373 MPa); however, this increase was mainly localised and associated with a stiffer restorative assembly. Importantly, stresses were redistributed away from critical regions such as cusp tips and cervical margins, which is biomechanically favourable despite higher peak values. Dentin stress values were comparatively lower and remained relatively consistent among all models (34.2 MPa). This suggests that none of the restorative strategies excessively transferred stress to dentin, thereby reducing the risk of dentinal crack initiation or root fracture. The stability of dentin stress in Model 4 indicates effective stress absorption by the reinforcing fibres rather than harmful stress concentration within the tooth structure. Similar findings were observed in a study by Navimipour et al. [17].

Interpretation of VM stress is critical for understanding these biomechanical outcomes. VM stress represents a combined measure of tensile, compressive, and shear stresses acting on a structure and is commonly used to predict regions susceptible to material yielding or failure [18]. Higher VM stress values indicate areas of stress concentration, which are clinically relevant as potential sites for crack initiation. When such stresses are localised within enamel, dentin, or cervical regions, they are more likely to result in unfavourable or non-repairable fractures [19]. Therefore, both the magnitude and the location of VM stress must be considered when assessing restorative performance.

Direct composite model (Model 1) exhibited the least favourable stress distribution, with distinct stress concentrations at the buccal and palatal cusp inclines and in the cervical region. These stress peaks indicate an increased susceptibility to tensile fracture under occlusal loading, consistent with previous studies that report MOD cavities reduce cuspal stiffness and increase fracture risk in posterior teeth restored solely with composite resin [20]. The concentration of stresses at internal line angles further supports the notion that direct composite alone is insufficient to mitigate cusp deflection or prevent crack initiation in large Class II defects.

Model 2 demonstrated improved mechanical stability by mechanically linking the buccal and palatal cusps with a glass fibre post. This splinting effect minimised cusp separation and efficiently redirected stresses away from vulnerable regions, such as marginal ridges and cervical areas. A recent systematic review has confirmed that horizontal posts can improve load transfer, limit cuspal deflection, and enhance fracture resistance in endodontically treated and structurally compromised posterior teeth [21]. The present study supports these findings, showing favourable redistribution of stresses across the coronal structure.

The incorporation of polyethylene fibre (Ribbond) in Module 3 resulted in a more uniform stress distribution and lower peak stress at the cusp tips. Acting as a stress-dampening layer, the fibre redistributed tensile forces within the composite and minimised localised stress concentrations. These observations align with previous reports that demonstrate polyethene fibres enhance fracture resistance and delay crack propagation in composite restorations [22]. Notably, the combined reinforcement technique in Module 4 exhibited the highest overall VM stress values (99.42 MPa) while demonstrating the lowest deformation (3.72 µm), indicating the formation of a stiffer restorative complex capable of effectively resisting deformation under occlusal loading. The elevated stress values in this model reflect efficient load-bearing rather than unfavourable stress concentrations, as stress localisation was predominantly confined to the enamel and restorative materials rather than the dentin or cervical regions. Clinically, this stress distribution is advantageous, as it reduces the risk of catastrophic tooth fracture and favours more repairable failure patterns. From a biomechanical perspective, stress localisation within enamel or restorative material may result in more favourable and repairable failure patterns, whereas stress transfer to dentin and cervical areas increases the risk of irreversible tooth fracture. The reduced cuspal deformation and redistribution of stresses away from structurally critical regions observed in the combined reinforcement model therefore suggest improved biomechanical behaviour despite the higher peak stress values. Similar observations have been reported in previous finite element studies evaluating fibre-reinforced restorations, where reinforcement strategies improved stress redistribution without necessarily reducing peak stress magnitude [17,21].

The synergistic incorporation of a horizontal glass fibre post and polyethylene fibre reinforcement provided both structural splinting and internal stress modulation, resulting in minimal stress concentration at critical regions, such as cusp tips, cervical margins, and internal line angles. Collectively, these findings indicate that the combined reinforcement approach offers the most favourable biomechanical behaviour among the tested restorative strategies [23]. These findings are consistent with earlier reports that combining fibre reinforcement methods provides superior stress control and reduced fracture risk compared to single-fibre strategies [24].

Previous in-vitro studies have shown that fibre reinforcement and fibre orientation do not significantly increase fracture resistance in root-filled premolars [25]. In agreement with these findings, the present FEA demonstrated that fibre reinforcement does not necessarily reduce peak stress values but plays a crucial role in redistributing stresses away from vulnerable tooth structures.

The present study has certain limitations. The FEMs assumed linear elastic and isotropic material properties for dental tissues and restorative materials. However, dentin and the periodontal ligament demonstrate anisotropic and viscoelastic behaviour, which may influence stress transfer and deformation patterns. Only static occlusal loading was applied in this analysis, whereas clinical conditions involve cyclic loading, temperature changes, and fatigue over time. Additionally, polymerisation shrinkage, interfacial degradation, and variability in fibre placement were not simulated. These factors could affect long-term restorative performance and fracture resistance.

Clinical significance

Extensive MOD cavities significantly compromise cuspal integrity and increase the risk of fracture when restored with composite resin alone. The findings of this study suggest that fibre reinforcement, either through polyethylene fibres or horizontal fibre posts, can improve stress distribution and reduce deformation under occlusal loads. The combined use of a horizontal post and fibre-reinforced composite demonstrated the most favourable biomechanical behaviour, offering enhanced resistance to stress concentration at critical regions of the tooth. Clinically, these reinforcement strategies may contribute to greater restoration longevity, improved cuspal stability, and reduced incidence of catastrophic fracture in posterior teeth with extensive structural loss.

## Conclusions

Within the limitations of this study, direct composite restorations showed unfavourable stress concentration in endodontically treated premolars with MOD cavities. Fibre reinforcement, using either horizontal fibre posts or polyethylene fibres, improved stress distribution and reduced cuspal deformation. The combined reinforcement technique demonstrated the most favourable biomechanical behaviour, suggesting that fibre-reinforced restorative strategies, particularly when used in combination, may serve as a conservative and effective alternative to conventional full-coverage restorations for structurally compromised, endodontically treated premolars.
